# Flexural Strength of Glass and Polyethylene Fiber Combined with Three Different Composites

**Published:** 2013-03

**Authors:** F Sharafeddin, AA Alavi, Z Talei

**Affiliations:** a Dept. of Operative Dentistry, Biomaterials Research Center, School of Dentistry, Shiraz University of Medial Sciences, Shiraz, Iran; b Dept. of Operative Dentistry, School of Dentistry, Shiraz University of Medial Sciences, Shiraz, Iran

**Keywords:** Glass Fiber, Poly Ethylene Fiber, Flexural Strength, Composite

## Abstract

**Statement of Problem: **The flexure of the fiber- reinforced composites (FRC) which can be generally used instead of fixed metal- framework prostheses have been more advocated due to the enormous demands for the conservative and esthetic restoration. The flexure of the fiber should be well-fitted to its covering composite. No study has been reported the comparison of the combination of glass and polyethylene fiber with particulate filled composite and fiber reinforced composite yet.

**Purpose:** This study compared the flexural strength of two types of fibers combined with three types of composites.

**Materials and Method:** Sixty-six specimens were prepared in a split mold (25×2×2 mm). The specimens were divided into six groups according to the type of resin and the fiber (N = 11): group 1: Z250 composite + Polyethylene fiber; group 2: Build It composite + Polyethylene fiber; group 3: Nulite F composite+ Polyethylene fiber; group 4: glass fiber + Z250 composite; group 5: glass fiber + Build-It composite and group 6: glass fiber + Nulite F. The mean ﬂexural strengths (MPa) values were determined in a 3-point bending test at a crosshead speed of 1 mm/min by a universal testing machine (Zwick/Roell Z020, Germany). The results were statistically analyzed, using one and two- way ANOVA and LSD post-hoc tests (*p*< 0.05).

**Results:** The highest flexural strength was registered for glass fiber in combination with Z250 composite (500 MPa) and the lowest for polyethylene fiber in combination with Build-It composite (188 MPa). One-way ANOVA test revealed that there was no statistically significant difference between polyethylene fiber combinations (*p*= 0.62) but there was a significant difference between glass fiber combinations (*p*= 0.0001). Two-way ANOVA revealed that the fiber type had a significant effect on flexural strength (*p*= 0.0001).

**Conclusion:** The choice of ﬁber and composite type was shown to have a signiﬁcant positive influence on the ﬂexural properties of the ﬁber-reinforced composite. Glass fiber has a significant influence on the flexural properties of directly- made specimens.

## Introduction

Recently, using Fiber-Reinforced Composites (FRC) which can be mostly used instead of the fixed metal framework prostheses, has been more advocated due to the enormous demands for the conservative and esthetic restoration. Compared with fixed metal dental prostheses, this type of restoration is lighter and more elegant. In addition, it can be attached to the dental tissues and will cause less harm to the remained teeth [1]. Although the durability of these types of prostheses is smaller than the metal frameworks, the cost and the time consumed to prepare such conservative prostheses are lesser [1]. FRC's durability has been reported differently in the related studies, so that the overall durability rate of 75 to 94.75 percent has been reported after three to five years [2-4]. 

FRC is a combination of fiber and resin matrix. Fiber is the reinforcing part, providing stability and stiffness, whereas resin matrix is the protecting part, producing the reinforcement and the ability to work with the material [5-6]. The mechanical characteristics and the effectiveness of the fiber reinforcement in FRC are based on the type of the fiber (Glass, Carbon, Polyethylene, Aramid), quantity of fibers, fiber structure including unidirectional, bidirectional and randomly oriented fiber, fiber position, fiber-resin matrix adhesion, fiber and resin matrix properties, the quality of fiber impregnation and water sorption of the FRC matrix [7-8]. 

The type of the fiber which is used to produce FRC depends on the purpose of its usage and its associated features and characteristics. In the laboratory, various types of fiberglass are employed, whereas in dental offices, the polymers reinforced by polyethylene are directly applied [9]. 

In a study, the composites filled with glass fibers showed high resistance against fracture or crack stopper and provided local support to eliminate the energy which was produced during the fracture. Reinforcing the composite by single, silicon-nitrate Whisker crystals was gained as well [10]. 

Moreover, as a veneer, the fiber-covering characteristics of the composite resins affect the physical and mechanical properties of the FRC and its esthetic characteristics [11]. Fiber works as a substructure and spreads the stresses produced by chewing, while the surface composite, provides its anatomic counter and beauty [12-13]. 

To achieve the high stiffness of the FRC, The flexure of the fiber should be well-fitted to its covering composite [12]. The Particulated Filled Composite (PFC), which is mostly presented as hybrid and microfill, can provide a durable intra oral restoration. Many studies have approached to find a way to improve the mechanical properties of PFCs. These include choosing a suitable filler and resin matrix, using different curing methods, reinforcing composite resin with micro-scaled fiberglass particles, and Whisker, using compacted porous ceramic fillers and improving the filler content [15]. Nonetheless, these substances do not have the adequate flexural strength to replace the lost tooth. Combination of PFC with FRC has already shown an improvement in mechanical properties when used in vivo [16-17]. Meanwhile, the possibility of using this combination directly and its attachment to the dental tissues has made it feasible to apply the compound (PCF with FRC) in order to fabricate the bridge and to substitute it for the lost tooth [18-20]. 

In this study, we analyzed glass fiber in direct application and compared its flexural strength with polyethylene fiber. Moreover, three composites were tested in combination with these two fibers. These include Z250 composite, which is a type of Particulated Filled Composites (PFC) and is reinforced by the blend of silica-zirconium, and Build It and Nulite F Composites, which are filled by fiberglass particles. The aim of this study was to investigate the flexural strength to achieve the desirable combination with high mechanical qualities. 

## Materials and Method

Details of the materials used in this experimental study are given in Table 1. A total of 66 specimens in 6 groups (n=11) was prepared in a split mold with 2 × 2 × 25 mm- slot dimensions in the center of the mold.

To assess the Flexural strength test, a brassy split mold was prepared, which has a slot for space with 2 × 2 × 25 (mm) dimensions. Sixty-six specimens with the mentioned dimensions were made in six groups, varying the type of the fiber and the composite resin used In groups 1 to 3, a 25mm-polyethylene fiber was used and soaked in Resist (BTD, Australia) in the saturated container for 20 minutes, away from light, to become fully impregnated with resin. Thereafter, it was taken out of the container by a single move and put at the bottom of the mold in its tensile side without holding the air, while the orientation of the fiber was always along the axis of the mold .Several laboratory studies demonstrated that the maximum flexural strength would be acquired when the fiber has been placed at the bottom of the specimen 18-19], as it was in this study. In groups 4, 5 and 6, the preimpregnated glass fibers were cut into strips to length of 25 mm and placed at the bottom of the mold. Then, three different types of composite resins: Z250, Nulite F and Built. It was carefully packed over the fibers. To remove excess materials and to avoid air entrapment; all specimens were pressed by a transparent strip and then a glass slab was placed on them. 

**Table 1:** Materials used in this study

**Materials**	**Manufacture**	**Chemical Composition**
Fiber ribbon	Angelus, Brazil	Glass fiber
Fiber braided	BTD, Australia	Polyethylene fiber
Z250 (light cure)	3M ESPE St. Paul	BIS-GMA,UDMA, BIS EMA
Nulite F (light cure)	BTD, Australia	Micro rod reinforced composite, hybrid BIS-GMA
Build It FR (dual cure)	Pentron Corp	Fiber reinforced core build up, glass filler, chopped, BIS GMA, UDMA
Resist (bonding)	BTD, Australia	Unfilled resin, low viscosity, BIS-GMA, UDMA, COMPHORQUINONE

Finally, the specimens were photopolymerized by a LED unit (LED, Demetron, Kerr) in four areas of the mold with the power of 1100 mW/cm^2^-light for 40 seconds. After the removal the specimens from the mold, dimensions were controlled by a caliper and the specimens were polished with a diamond bur. Prior to the performance of the test, the specimens were stored at room temperature in distilled water for 24 hours. All procedures were carried out by one operator in order to standardize the procedure.

**Flexural Strength Test**

To measure the flexural strength, specimens were tested on Universal Testing Machine at a crosshead speed of 1mm/min under the Three Point Bend Testing. 

The values obtained from specimens’ fracture which was (in Newton) were modified into the following formula (in MPa) [7]: 

δ = 3ωI / 2bd^2^ Where 

δ = Flexural Strength

ω = Maximum load applied to the specimen in Newton

I = Distance between two supports in millimeters

b = Width of the specimen in millimeters and

d = Height of the specimen in millimeters.

The data collected from the fracture were tabulated and analyzed by SPSS, using one-way ANOVA and two-way ANOVA and LSD Post-hoc tests (*p*< 0.05).

## Results

The highest flexural strength was recorded for glass fiber in combination with Z250 composite (500 MPa) and the lowest for polyethylene fiber in combination with Build It composite (188 MPa) (Table 2).

**Table 2:** Flexural strength and p value of specimens

**Composite **	**Polyethylene ** **combination**		**Glass ** **combination**		**P value**
	**SD**	**Mean (MPa)**		**Mean ** **(MPa)**	
Nulite F	33.22	203.90	.009	243.34	Nulite F
Build It	55.10	188.09	.0001	331.09	Build It
Z250	39.38	203.45	.0001	500.09	Z250

The One- Way Anova analysis revealed that there was no significant difference between polyethylene fiber combinations (*p*= 0.62) but there was a significant difference between glass fiber combinations (*p*= 0.0001) (Table 3). 

The Two- Way Anova revealed that fiber type had a significant effect on flexural strength (*p*= 0.0001). 

The LSD test showed that there was no substantial difference between Nulite F and Built It composites, although the flexural strength of Z250 was significantly greater than that of the other two composites.

**Figure 1:**
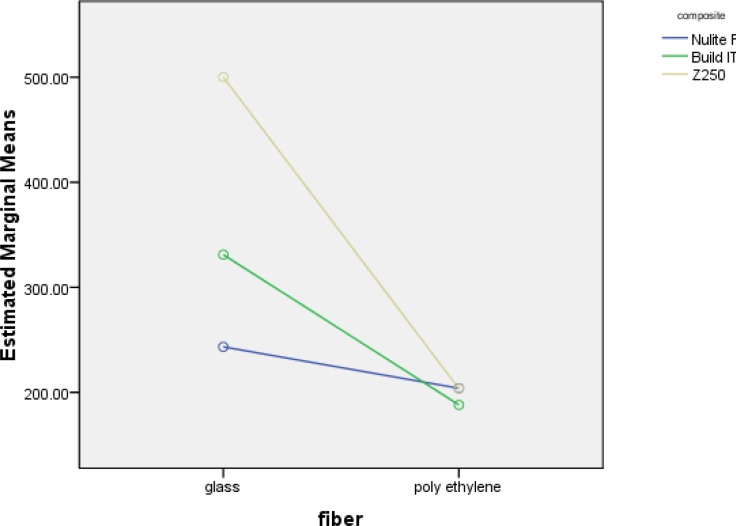
Estimated marginal means of flexural strength

Figure1 shows that the flexural strength of the three test- groups decreased with different rates.

## Discussion

Flexural strength and elasticity modulus are the two most important mechanical characteristics in the evalua-tion of the fiber reinforcement systems. The flexural strength test, which is performed by three- point- bending test method, measures both tensile and compressive states at the same time [1, 21].

**Table 3:** Flexural strength and p value of specimens

**Fiber**	**Nulite F**		**Build-It**		**Z250**		**P value**
	**Mean** **) ** **MPa)**	**SD**	**Mean** **) ** **MPa)**	**SD**	**Mean** **) ** **MPa)**	**SD**	
Glass	243.34	500.09	331.09	79.19	500.09	31.24	.0001
Polyethylene	203.90	203.45	188.09	55.10	203.45	39.38	.629

This study showed that different fiber types when combined with the given composites significantly differ in their flexural strengths and the whole compositions with fiberglass have more flexural strength. 

It seems that one of the reasons that the applied fiberglass in this study had more strength than the polyethylene fiber was its preimpregnation when manufactured. Pre-impregnation improves the bonding properties of the fiber and creates a homogenous substance, which in turn, increases the strength 2 to 3 times more than manually impregnated fibers [21]. An investigation stated that fiberglass provided an excellent adhesion between the fiber and resin matrix and reinforcing effects of fiberglass increased the mechanical qualities of the matrix [7]. 

In this study, no difference was found between the different combinations of the composites and polyethylene fiber. This means that the flexural strength of different types of composites combined with polyethylene fiber is approximately the same. However, the findings showed that there was a remarkable difference between the flexural strengths of various types of those composites when combined with fiberglass. The results displayed that the difference in average flexural strengths of the fiberglass when combined with three types of composites Nulite F, Built It and Z250 was significant. Moreover, it revealed that a combination of fiberglass with Z250 had flexural strength greater than the other two composites. This could be partially attributed to the differences in filler load, filler type, resin matrix and its composition.

 Glass is chemically an amorphous substance and contains tetra- hydra- silica. These substances have randomly combined with each other in a network. Consequently, glass fibers have different chemical and physical characteristics which distinct them from organic fibers such as ultra high molecular weight polyethylene (UHMWPE) and Kevlar [8]. Improved adhesion of composites and glass fibers could be due to the silica contents of the fiber and consequent stronger bonds which in turn lead to an increased flexural strength. [22]. 

 Another reason for the high flexural strength of the fiberglass and Z250 composite resin combination could be the strongest chemical bond between the fiberglass and the dental polymers such as Methyl Metacrylate and Bis-GMA or UDMA in Z250. While Nulite F contains only Bis-GMA with 71% of filler in volume sized 0.04 to 16 microns. Z250 contains 60% of filler particles in volume including zirconium- silica in tiny sizes (average 0.6 microns). The combination of Built It composite with glassfiber presented greater flexural strength than glassfiber and Nulite F compound. This could be due to the presence of UDMA and Bis-GMA together with glassfiber in a "Chopped" form [23], which increases the compressive strength of the substance, and can affect the flexural strength. The ratio of filler to resin is also important and since the amount of the filler is higher than resin, the light penetration will be more difficult during curing [24]. 

Therefore, a composite resin may not achieve its ultimate strength and this might explain the reason of the lower strength of Nulite F compared with Z250. Even though Built It, a dual cure composite contains a high rate of filler, when curing light is insufficient, this composite will become chemically hardened after 4 minutes and achieves its ultimate strength. It seems the problem involving this composite is its high shrinkage during the hardening process and the stress which is created during polymerization. 

In a similar study, it was also found that the fractural resistance of Fixed Partial Denture (FPD) highly increases with glassfiber frame. Moreover, the reinforcing effect of the fiber extremely depended on the type of the composite resin used [25-26]. 

Eronate et al. reported that the flexural strength of the hybrid composite combined with glassfiber was greatly higher than that of the combination of the glassfiber with microfill composite [27]. This finding is in accordance with the results achieved in the present study. In addition to the type of the fiber, it appears that the type of the composite can also influence the flexural strength of the fiber reinforced composites. 

In other research, polyethylene fiber did not increase the flexural strength of the hybrid composites [1]. The results were in line with the results of the present study. 

Our study entailed that the use fiberglass can improve and increase the strength of the specimen made by this type of fiber. This type of fiber embraces beauty as well as strength so that it can be conveniently used to replace a lost tooth in the anterior regions [6]. 

Eronate et al*. *reported that the degree of impregnation of the fiber used for reinforcement affects its characteristics. When the degree of impregnation is not enough, some voids are created in the polymer matrix. This will decrease the mechanical characteristics such as flexural strength in FRC. This will also cause water absorption in FRC and in long terms affects the consistency of FRC in the moist oral cavity [27]. Resist , an unfilled resin based on Bis-GMA and UDMA, is the bonding agent recommended for impregnation of polyethylene fiber. It seems that the Resist provides the adhesion between the fibers and also diminishes the stress transferred from the matrix to the polyethylene fiber. When remarkable amount of the Resist remains around the fiber, the stability of FRC decreases. In one study Tushima et al. found that using a bonding agent without filler produces less flexural strength than the bonding agent with filler. It was the resin which contained filler and allowed a suitable fiber wetting with a maximum reinforcement. The bonding solvent compatibility with the composites was definitely a very important factor affecting flexural strength [28]. Hypothetically, if the bonding resin contains filler, the shrinkage will be reduced during the polymerization and therefore, produces less stress in the bonded increment [11, 29]. In our study, using Resist for impregnating polyethylene fiber, apparently produced stress and consequently weakened the fiber.

In laboratory studies, most of the fractures in the FRC bridges occur in the distance between the fibers and resin matrix, forcing location, pontic, and the pontic- abutment connectors [30]. In this study, in the groups with fiberglass as the substructure of the composites, most of the fractures separated in two complete parts. However in the groups with polyethylene fiber, polyethylene fiber prevented the complete separation of the two parts. In the groups with polyethylene fiber, the parts were attached to the fiber throughout the test .As long as the force was applied, until the head of the force-applying device moved the bent parts out of their support by pressure. So, in the specimens made with fiberglass, the fractures occurred frequently when the brittle design and the complete fracture turned out after exerting an excessive force. 

The findings of this study are in accordance with Pereira et al ‘s study in which the specimens, after force insertion, have been separated in two discrete parts in the veneer area of the composite while still attached to each other by the intact polyethylene fiber, this showed the stability and firmness of the polyethylene structure [1]. 

 In this study, the average fracture force for specimens with polyethylene fiber was reported 203, 188, and 203 MPa. This was definitely lower than the force endured by the made-with-glassfiber specimens that were reported as 243,331 and 500 MPa. 

It comes into sight that more clinical and long term studies are required to encompass laboratory limitations to achieve more relevant results. 

## Conclusion

Within the limits of this in vitro study, it is possible to conclude that:

1. The type of the fiber has a great influence on the flexural strength of specimens.
2. Using preimpregnated glass fiber can directly enhance flexural property.
3. Different Composite types when combined with glass fiber affect flexural strength but do not affect the flexural strength when combined with polyethylene fiber.
4. Glass fiber-Z250 combination has the highest flexural strength.
5. Glass fiber has a brittle failure type but polyethylene fiber prevents complete separation of fractured specimens.
